# The role of hormonal therapy in patients with relapsed high-grade ovarian carcinoma: a retrospective series of tamoxifen and letrozole

**DOI:** 10.1186/s12885-017-3440-0

**Published:** 2017-06-30

**Authors:** Angela George, Jennifer McLachlan, Nina Tunariu, Chiara Della Pepa, Cristina Migali, Martin Gore, Stan Kaye, Susana Banerjee

**Affiliations:** 1 The Royal Marsden NHS Foundation Trust and Division of Clinical Studies, Institute of Cancer Research, Fulham Road, London, SW3 6JJ UK; 2 Radiology Department, The Royal Marsden NHS Foundation Trust, 203 Fulham Road, London, SW3 6JJ UK

**Keywords:** Endocrine therapy, Hormonal therapy, Letrozole, Ovarian cancer, Tamoxifen

## Abstract

**Background:**

Hormonal therapy is used as a treatment option in high-grade ovarian carcinoma (HGOC), but the role and choice of treatment remains unclear. Agents used include tamoxifen and aromatase inhibitors. Our aim was to evaluate the efficacy of tamoxifen (T) and letrozole (L) in HGOC in clinical practice and investigate factors influencing clinical outcome.

**Methods:**

A retrospective review of patients with relapsed HGOC treated with either tamoxifen or letrozole at the Royal Marsden Hospital between 2007 and 2012 was performed. The primary endpoint of the study was objective response rate (ORR). Secondary endpoints included CA125 response, clinical benefit rate (CBR) and duration of response. Platinum-sensitivity and ER-status were evaluated as predictors of treatment response.

**Results:**

97 patients were included (43 T, 54 L); median age 63 years (20–92); 91% high-grade serous; median number of lines of prior chemotherapy 3 (1–8); 60% platinum-resistant, 40% platinum-sensitive; 52% ER + ve, 1% ER-ve, 47% unknown. 14 patients (6 T, 8 L) achieved a partial response, with ORR (RECIST) of 14% (T) and 15% (L). The CBR for ≥3 months was 65% (22/43) for tamoxifen and 56% (22/54) for letrozole. There was no significant difference in ORR (*p* = 0.99) or CBR (*p* = 0.14) between tamoxifen and letrozole. 22 patients (23%) had a CA-125 response with hormonal therapy (10 T – 23% and 12 L – 22%). ORR did not differ by platinum sensitivity (*p* = 0.42); or ER-status (positive vs unknown, *p* = 0.12). Responders to letrozole had longer durations of response than responders to tamoxifen (26 vs 11.5 months, *p* = 0.03), but equivalent disease stability duration (9.6 vs 7.2 months respectively, *p* = 0.11).

**Conclusions:**

Within the constraints of a retrospective study, we identified that patients treated with letrozole had a significantly longer duration of response than those treated with tamoxifen. Treatment with either tamoxifen or letrozole is a rational treatment option for patients with ER + ve HGOC, with equivalent ORR, CBR and disease stability.

## Background

Epithelial ovarian cancer is the seventh most common cancer in women and accounts for the highest mortality of all the gynaecological malignancies [1]. Unfortunately, the majority of women with advanced disease relapse following primary debulking surgery and platinum-taxane chemotherapy, and ultimately die from progressive disease. Repeated courses of systemic chemotherapy may result in disease remission for many patients, but these episodes are typically characterized by progressively shorter progression-free intervals and often accumulating toxicity, highlighting the importance for better approaches to treating recurrent disease.

Hormonal therapy (has also been referred to as hormone therapy, endocrine therapy) is commonly used as a treatment option in patients with recurrent ovarian cancer who have exhausted or are not suitable for further standard lines of systemic chemotherapy. Hormonal therapy has also been postulated to be effective in the setting of relapsed disease with a rising CA125 before the onset of significant clinical symptoms [2]. Hormonal therapy provides an attractive therapeutic option in these patients due to the convenience of oral administration, and generally tolerable safety profile compared to chemotherapy and other targeted therapies. Although there is a strong evidence base to support the use of hormonal therapy in the treatment of early and metastatic estrogen receptor (ER) -positive breast cancer, there are few prospective clinical trials evaluating this approach in recurrent ovarian cancer.

The rate of ER-positivity in epithelial ovarian cancer is reported to be 43–81%, depending on the definition and methodology used [3–6]. Pre-clinical models have demonstrated that ovarian cancer cells that express high levels of estrogen receptors are stimulated by estrogens and inhibited by anti-estrogens, providing rationale for the use of hormonal therapy in this disease [7–9].

Tamoxifen, a selective estrogen receptor modulator (SERM), is a competitive inhibitor of estrogen binding to the ER. The response rate to tamoxifen in small phase II studies of patients with recurrent ovarian cancer ranges between 3.2–17.1% (Table 1) [10–12]. A Cochrane Database Systematic Review of tamoxifen for recurrent ovarian cancer including 623 patients demonstrated a 10% objective response rate and a 32% disease stabilisation rate [13]. Letrozole is a potent non-steroidal aromatase inhibitor which suppresses plasma estrogen levels by inhibiting or inactivating aromatase, the enzyme responsible for the peripheral conversion of androgens to estrogens. The response rate to letrozole in small phase II studies of patients with recurrent ovarian cancer ranges between 0 to 15% (Table 1) [14–17].

**Table 1:** Published trials of hormonal therapy in ovarian cancer

Author [Ref]	Treatment	Study population	Phase	N	ORR (%)	CR (%)	PR (%)	SD (%)	CBR (%)
Argenta, et al., 2009 [18]	Fulvestrant	Recurrent ER+ OC	II	26	0	0	0	50	50
Ramirez, et al., 2008 [14]	Letrozole 2.5 mg OD	Recurrent ER+ high-grade OC, platinum- and taxane-resistant	II	33 (31 evaluable by RECIST)	3	0	3	23	26
Smyth et al., 2007 [15]	Letrozole 2.5 mg OD	Recurrent ER+ OC	II	44 (33 evaluable by RECIST)	9	0	9	42	51
Wagner, et al., 2007 [19]	Tamoxifen 40 mg OD + gefitinib 500 mg OD	Recurrent OC, platinum- and taxane resistant	II	56 (49 evaluable by RECIST)	0	0	0	32.7	32.7
Papadimitriou, et al., 2004 [16]	Letrozole 2.5 mg OD	Recurrent OC	II	27 (21 evaluable by WHO criteria)	15	5	10	19	29
del Carmen, et al., 2003 [20]	Anastrozole 1 mg OD	Recurrent OC	II	53	1.9	0	1.9	42	43.9
Bowman, et al., 2002 [17]	Letrozole 2.5 mg OD	Recurrent OC	II	60 (50 evaluable by UICC criteria)	0	0	0	17	17
Hatch, et al., 1991 [12]	Tamoxifen 20 mg BD	Recurrent OC	II	105	17.1	9.5	7.6	38	45.6
Weiner, et al., 1987 [10]	Tamoxifen 10 mg BD	Recurrent OC	II	37 (31 evaluable)	3.2	3.2	6.4	19.3	28.9
Schwartz, et al., 1982 [11]	Tamoxifen 20 mg OD	Recurrent OC	II	13	7.4	0	7.7	30.8	38.5

*Abbreviations: BD* twice daily, *CBR* clinical benefit rate, *CR* complete response, *ER* estrogen receptor, *OC* ovarian cancer, *OD* once daily, *ORR* objective response rate, *PD* progressive disease, *PR* partial response, *SD* stable disease

Studies were selected using a pubmed literature search using search terms “ovarian cancer” and “letrozole”, and “ovarian cancer” and “tamoxifen”, as well as a search for studies using clinicaltrials.gov using search terms “hormonal therapy” and conditions “ovarian cancer”.

The choice of hormonal treatment in recurrent high-grade ovarian carcinoma (HGOC) remains unclear. It has been suggested that response to these agents is associated with ER-status [15, 17]. A study by Smyth et al. reported an almost doubling of CA125 response rate for letrozole when patients with recurrent ovarian cancer were pre-selected for expression of the ER (9% in unselected vs 17% in ER+ patients) [15, 17]. Our aim was to assess the efficacy of tamoxifen and letrozole in HGOC and explore potential factors influencing clinical outcome.

## Methods

This study was approved by the Royal Marsden Clinical Research Committee, and the need for informed consent was waived, as this was a retrospective study of clinical practice. All women with HGOC treated with tamoxifen or letrozole between June 2007 and June 2012 at the Royal Marsden Hospital, with measurable disease by Response Evaluation Criteria in Solid Tumours (RECIST) 1.1, were identified using electronic patient records. Patients with low-grade tumours were excluded from the case series. The primary endpoint of the study was objective response rate (ORR). Secondary endpoints included clinical benefit rate (CBR), CA125 response and duration of response. Platinum-sensitivity and ER-status were assessed as predictors of treatment response.

Data including baseline patient characteristics, ER- and progesterone-receptor (PR) status, platinum-sensitivity status, hormonal treatments used, CA125 response to treatment, best radiological response to treatment, date of radiological progression and date of death or last follow-up were retrieved from the electronic patient records.

### Statistical methods

The best overall response to hormonal treatment (complete response, partial response, stable disease or progressive disease) was defined according to RECIST 1.1 and reviewed by a specialist gynae-oncology radiologist. CBR was defined as the percentage of patients with a response (complete and partial) or stable disease for ≥3 months. CA125 response was defined according to Gynaecologic Cancer Intergroup (GCIG) criteria. Patients alive at the time of evaluation and those with loss of follow-up were censored at last follow-up. Imaging assessment was performed three months after initiating treatment to assess response and three-monthly thereafter unless there were clinical reasons to scan sooner.

## Results

A total of 97 patients were included in the study, all of whom were patients who were either considered to have no other chemotherapeutic/trial options, those for whom further chemotherapy was contraindicated due to poor performance status or patient refusal. Baseline characteristics of the patients are summarized in Table 2. The median number of prior lines of chemotherapy was 3. Forty-three patients were treated with tamoxifen and 54 patients were treated with letrozole. There were 17 patients who received tamoxifen and letrozole (as single agents) during the disease course. For this group of patients, the first hormonal therapy was considered for response analysis. All patients treated with letrozole received a dose of 2.5 mg daily. Of those that were treated with tamoxifen, 36 (83.7%) received 20 mg daily and 7 (16.3%) received 40 mg daily. The choice of tamoxifen or letrozole was at the discretion of the clinician and took into consideration the risks of toxicities. For example, patients with clinically significant osteoporosis were treated with tamoxifen rather than letrozole.

**Table 2:** Baseline characteristics (*n* = 97)

	N (%)
Median age at diagnosis (range)	63 (20–92)
Histological subtype	
High-grade serous	90 (92.7)
High-grade endometrioid	5 (5.2)
Clear cell	2 (2.1)
FIGO stage at diagnosis	
I	7 (7.2)
II	4 (4.1)
III	73 (75.3)
IV	13 (13.4)
ER-status	
Positive	50 (51.5)
Negative	1 (1)
Unknown	46 (47.4)
PR-status	
Positive	20 (20.1)
Negative	11 (11.3)
Unknown	66 (68)
Primary Surgery status	
Complete debulking	37 (38.1)
Suboptimal debulking (>1 cm residual disease)	44 (45.3)
No surgery	16 (16.5)
Platinum-sensitivity	
Platinum-sensitive	39 (40)
Platinum-resistant	58 (60)
Number of lines of prior chemotherapy	
1	18 (18.6)
2	15 (15.5)
3	21 (21.6)
4	17 (17.5)
5	10 (10.3)
≥ 6	16 (16.5)

### Efficacy

**Fig. 1:**
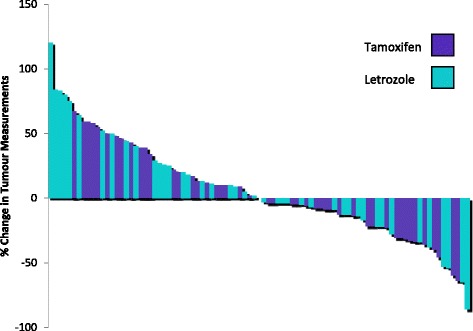
Waterfall plot of percentage change in tumour measurements

Radiological and CA125 response to hormonal therapy is summarized in Table 3. There were no patients with a complete response. In total, 14 patients had a radiological partial response to hormonal therapy, with an ORR of 14.4%. Six patients had a partial response to tamoxifen (ORR 14%) and 8 patients had a partial response to letrozole (ORR 14.8%). 22 patients treated with tamoxifen and 22 patients treated with letrozole achieved stable disease for greater than 3 months, with a CBR of 65.1% in the tamoxifen cohort and 55.6% in the letrozole cohort. There was no significant difference in ORR (*p* = 0.99) or CBR (*p* = 0.14) between the tamoxifen and letrozole cohorts. Percentage change in tumour measurements with hormonal treatment for each patient are demonstrated in Fig. 1. Amongst the patients that had a response following hormonal therapy, the median duration of response was longer in patients treated with letrozole compared with those treated with tamoxifen (26 vs 11.5 months, *p* = 0.03), but there was no significant difference in the median duration of disease stabilisation between the letrozole and tamoxifen treated patients (9.6 vs 7.2 months respectively, *p* = 0.11).

**Table 3:** Response to hormonal therapy

Treatment	PR (%)	SD (%)	PD (%)	ORR (%)	CBR (%)	CA125 response (%)
Tamoxifen (*n* = 43)	6 (14)	22 (51.2)	15 (34.9)	6 (14)	28 (65.1)	10 (23.3)
Letrozole (*n* = 54)	8 (14.8)	22 (40.7)	24 (44.4)	8 (14.8)	30 (55.6)	12 (22.2)
Total (*n* = 97)	14 (14.4)	44 (45.4)	39 (40.2)	14 (14.4)	58 (59.8)	22 (22.7)

Abbreviations: CBR clinical benefit rate, ORR objective response rate, PD progressive disease, PR partial response, SD stable disease

**Fig. 2:**
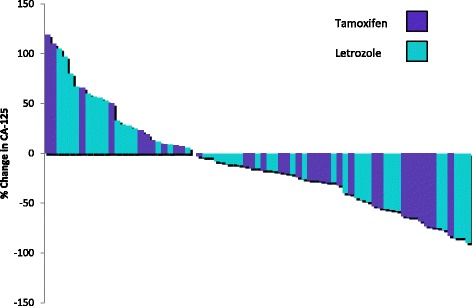
Waterfall plot of CA125 response

In total, 22 patients (22.7%) had a CA125 response; 10 patients (23.3%) in the tamoxifen group, and 12 patients (22.2%) in the letrozole group. Percentage change in CA125 values with hormonal treatment for each patient are demonstrated in Fig. 2.

For those patients in which both radiological status and CA-125 response was evaluable (*n* = 22), responses were concordant. All patients with a CA-125 response had either a RECIST partial response or stable disease.

### Predictive markers of response

Of those 14 patients who achieved a partial response, 7 (50%) had tumours that were ER + ve, and in 7 (50%), the ER-status was unknown. Seven patients (50%) had platinum-sensitive disease and 7 (50%) had platinum-resistant disease. ORR did not differ by ER status (positive vs unknown, *p* = 0.12); or platinum sensitivity (*p* = 0.42). The ORR to hormonal therapy for patients with platinum-sensitive disease was 17.9% (7/39) and 12.1% (7/58) for those with platinum-resistant disease. There was no significant difference between the ORR following tamoxifen or letrozole in relation to platinum-sensitivity status (Letrozole: platinum-resistant 13%; platinum-sensitive 17% Tamoxifen: platinum-resistant 11%; platinum-sensitive 17%).

Assessment of response by histological subtype was limited by the small numbers of non–serous histology in this cohort of patients with high grade ovarian cancer. However, of the five patients with high grade endometrioid subtype, three derived a partial response and two had stable disease. Of the two patients with clear cell ovarian carcinoma, one had disease stabilisation and the other disease progression.

### Subsequent therapy

There were 17 patients who received both letrozole and tamoxifen sequentially after progression on the first hormonal treatment. Whilst only the first response was considered in the overall statistical analysis, nine patients had prolonged disease stabilisation with the second endocrine agent. The best response for patients whom received letrozole subsequent to tamoxifen (*n* = 17) were as follows: 0 partial response, 9 (53%) stable disease, 6 (35%) progressive disease and 2 (12%) intolerant to therapy. The best response for patients whom received tamoxifen subsequent to letrozole (*n* = 17) were as follows: 2 (12%) partial response, 13 (76%) stable disease, 2 (12%) progressive disease.

At time of censoring, more than half (50, 52%) of the patients had been treated with one or more subsequent lines of systemic treatment, including both chemotherapy and Phase I trial agents. The details of numbers of subsequent lines of treatments are shown in Table 4.

**Table 4:** Number of lines of subsequent systemic treatments

Number of treatments	Tamoxifen patients	Letrozole patients
0 (*n* = 47)	22	25
1 (*n* = 28)	13	15
2 (*n* = 16)	6	10
3 (*n* = 4)	0	4
4 (*n* = 2)	2	0

## Discussion

In this retrospective series of women with recurrent HGOC, the ORR to hormonal therapy was 14.4%, with no significant difference in response observed between the tamoxifen and letrozole groups. However, patients treated with letrozole had a significantly longer duration of response than those treated with tamoxifen. This ORR is at the higher end of the range of responses reported in previous phase II trials of letrozole and tamoxifen in patients with recurrent ovarian cancer (Table 1). The ORR to letrozole in previous studies ranges from 0 to 15% [2, 14, 16, 17], and tamoxifen 3.2–17.1% (Table 1) [10–12]. The relatively high response rate in our series is encouraging given that the patient cohort included a heavily pre-treated population with more than a quarter of patients having received 5 or more lines of prior chemotherapy. There was a high proportion of patients with high-grade serous histology in the current study (91%). A large study examining ER-expression reported by the Ovarian Tumor Tissue Analysis Consortium demonstrated that 81% of 1742 high-grade serous carcinomas were ER-positive [3]. Although, there was no significant relationship between ER-status and ORR seen in this case series of 97 patients, it is important to note that information on ER-status was unavailable in 47% of patients.

The CBR reported in this study was almost 60%, suggesting that hormonal therapy provides a valid treatment option in women with recurrent ovarian cancer to maintain disease stability. Recurrent ovarian cancer remains an incurable disease and therefore stabilisation of disease should be valued as an important treatment outcome, particularly in heavily pre-treated patients who have exhausted standard lines of therapy or who wish to delay another line of chemotherapy taking into consideration quality of life. It is important to note that more than half of the patients went on to receive further lines of systemic treatment- in many cases due to stabilisation of symptoms and improvement in performance status. The majority of patients did not have significant toxicities whilst on letrozole or tamoxifen allowing these patients to have the opportunity of a further treatment option with potentially less detrimental impact on their quality of life than perceived with chemotherapy.

A major strength of this study is the large number of patients, which is relatively high compared to previous studies of hormonal therapy in recurrent ovarian cancer. Notably, there are several weaknesses, which mainly relate to the retrospective nature of this work. For example, information on toxicity was not consistently reported over the time period and therefore not collected. Toxicity and the potential impact on quality of life are important factors to take in to account when considering a treatment option, particularly in patients with heavily pre-treated disease. Previous studies have demonstrated that tamoxifen and letrozole are well-tolerated in patients with recurrent ovarian cancer [2, 14, 16, 17, 21]. Gastrointestinal side-effects (nausea and vomiting) are the most commonly reported adverse events with tamoxifen, and importantly there is also an increased risk of venous thromboembolism (1–2%) [22, 23]. The most commonly reported toxicities with letrozole in recurrent ovarian cancer include hot flushes (34%), myalgia/arthralgia (23%), headache (20%), nausea (16%) and fatigue (16%) [2].

A further weakness of the current study is the lack of information on ER-status in 47% of patients, which made it difficult to assess ER-status as a predictive marker of treatment response. ER-status was not routinely tested throughout the time period in question (2007–2012). It is now standard practice to assess ER by immunohistochemistry on all epithelial ovarian cancers at our institution. It would be worthwhile to explore further the significance of ER-status as a predictor of response to hormonal therapy in future studies. Previous studies of letrozole in recurrent ovarian cancer have demonstrated significant associations between high ER expression and radiological disease stabilisation and CA125 response [2, 17]. Bowman et al. reported significantly higher ER values in the group who achieved radiological stable disease with letrozole compared with those whose best response was progressive disease (*p* = 0.027). Similarly, those who achieved a CA125 response/stable disease had significantly higher ER, compared to those with CA125 progression (*p* = 0.013) [17]. Following this study, Smyth et al. reported an almost doubling of CA125 response rate for letrozole when patients with recurrent ovarian cancer were pre-selected for expression of the ER (9% in unselected patients vs 17% in ER+ patients) [15, 17].

This study did not show a statistically significant association between platinum-sensitivity and ORR although numerically, the response rate was slightly higher for platinum-sensitive cases (17.9%) than for platinum-resistant high grade ovarian cancer patients 12.1%. Previous studies have postulated that there may be an association between platinum-sensitivity and response to letrozole [14]. Ramirez et al. evaluated letrozole in patients with platinum-resistant ovarian cancer, reporting an ORR of 3%. In contrast, Papadimitriou et al. evaluated letrozole in a cohort of patients with recurrent ovarian cancer including 67% with platinum-sensitive disease, with an ORR of 15% [16]. The impact of platinum-sensitivity on clinical outcomes following hormonal therapy may warrant further exploration in a larger series.

The PARAGON (ANZGOG-0903) trial is a phase 2 ‘umbrella’ study of the aromatase inhibitor, anastrozole, in a number of groups of patients with estrogen or progesterone receptor positive recurrent/metastatic gynecological cancer [24]. This study included a group of asymptomatic ovarian cancer patients with GCIG-defined CA-125 progression and low-volume disease following first line treatment (*n* = 54). Preliminary results indicate a CBR at 3 months of 33%. Of note, 7 patients (7.7%) remained on anastrozole for more than 6 months. The results of the platinum-resistant/refractory group within the PARAGON trial are awaited.

It is important to note that endocrine agents other than anti-estrogens and aromatase inhibitors are being explored in recurrent ovarian cancer. It is well established that androgen receptors (AR) are commonly expressed in epithelial ovarian cancer [25], and novel agents targeting the AR signaling pathway are being investigated in this disease. Abiraterone, a CYP17 inhibitor, which irreversibly inhibits androgen biosynthesis, has recently been evaluated in a phase II study of 42 patients with recurrent epithelial ovarian cancer (CORAL study) [26]. 69% of patients were AR + ve using a cut-off value of 10%. Although the ORR to abiraterone in this study was low (2%), a subset of patients achieved sustained clinical benefit (CBR 26%) and detailed characterization of these patients is underway. Similarly, a phase II study of enzalutamide, an androgen receptor inhibitor, in patients with recurrent AR + ve ovarian cancer is currently recruiting patients (NCT01974765).

There are a number of ongoing trials exploring combinations of hormonal agents with other therapeutic agents in ovarian cancer. For example, a phase II single arm study of letrozole and everolimus, an mTOR inhibitor, in patients with platinum-resistant/refractory ovarian cancer or endometrial cancer is currently recruiting patients (NCT0218850). In addition, a phase II, open-label study of letrozole and ribociclib (CDK4/6 inhibitor) in patients with relapsed ER-positive ovarian cancer and endometrial cancer is underway (NCT02657928).

## Conclusions

In conclusion, our results demonstrate that hormonal therapy with either tamoxifen or letrozole is a valid treatment option for patients with recurrent HGOC, and has the potential to maintain disease stability for a prolonged period. There is a strong rationale for this approach and further research in this area should be conducted. More clinical trials are needed in this area, in particular phase III trials of hormonal therapy compared to other systemic agents.

## Abbreviations

AR: androgen receptor
CBR: clinical benefit rate
ER: estrogen receptor
HGOC: high-grade ovarian carcinoma
ORR: objective response rate
RECIST: Response Evaluation Criteria in Solid Tumours
